# Fruit size control by a zinc finger protein regulating pericarp cell size in tomato

**DOI:** 10.1186/s43897-021-00009-6

**Published:** 2021-08-14

**Authors:** Fangfang Zhao, Jiajing Zhang, Lin Weng, Meng Li, Quanhua Wang, Han Xiao

**Affiliations:** 1 National Key Laboratory of Plant Molecular Genetics, CAS Center for Excellence in Molecular Plant Sciences, Institute of Plant Physiology and Ecology, Chinese Academy of Sciences, Shanghai, 200032 China; 2 University of Chinese Academy of Sciences, No.19(A) Yuquan Road, Shijingshan District, Beijing, 100049 China; 3 Life and Environment Science College, Shanghai Normal University, No.100 Guilin Rd, Shanghai, 200234 China

**Keywords:** Zinc finger protein, Endoreduplication, Cell cycle, Cell size, Fruit size, Tomato (*Solanum lycopersicum*)

## Abstract

Fruit size is largely defined by the number and size of cells in the fruit. Endoreduplication – a specialized cell cycle – is highly associated with cell expansion during tomato fruit growth. However, how endoreduplication coupled with cell size is regulated remains poorly understood. In this study, we identified a zinc finger gene *SlPZF1* (*Solanum* *lycopersicum* *PERICARP-ASSOCIATED* *ZINC* *FINGER PROTEIN* 1) that was highly expressed in the pericarp of developing fruits. Plants with altered *SlPZF1* expression produced smaller fruits due to the reduction in cell size associated with weakened endoreduplication. Overexpressing *SlPZF1* delayed cell division phase by enhancing early expression of several key cell cycle regulators including *SlCYCD3;1* and two plant specific mitotic cyclin-dependent protein kinase (*SlCDKB1* and *SlCDKB2*) in the pericarp tissue. Furthermore, we identified 14 putative SlPZF1 interacting proteins (PZFIs) via yeast two hybrid screening. Several PZFIs, including Pre-mRNA-splicing factor (SlSMP1/PZFI4), PAPA-1-like conserved region family protein (PZFI6), Fanconi anemia complex components (PZFI3 and PZFI10) and bHLH transcription factor LONESOME HIGHWAY (SlLHW/PZFI14), are putatively involved in cell cycle regulation. Our results demonstrate that fruit growth in tomato requires balanced expression of the novel cell size regulator *SlPZF1*.

**Supplementary Information:**

The online version contains supplementary material available at 10.1186/s43897-021-00009-6.

## Core

*SlPZF1* encodes a member of C_2_H_2_ zinc finger protein family, preferentially expressed in the pericarp during tomato fruit development. Functional analysis reveals that *SlPZF1* controls fruit size through its action on cell size regulation, which is likely mediated by its interacting partners of cell cycle regulators. *SlPZF1* may be a good candidate of pericarp markers for dissecting the molecular mechanism underlying cell size regulation mediated by cell cycle in freshly fruits.

## Background

Tomato (*Solanum lycopersicum*) is one of the most important vegetable crops cultivated world-wide, which provides a rich source for human diets of vitamins, fiber, minerals and healthy compounds such as lycopene and carotenoids. The fleshy fruit of tomato is also a widely used model for fruit development study including fruit shape and size, ripening and metabolism. The growth of the fruit which is developed from pollinated ovary can be divided into four main stages including fruit set, cell division, cell expansion and ripening in tomato (Gillaspy et al. 1993). The fruit changes rapidly in size after pollination, for example, the number of pericarp cell layer almost doubles and the size of some mesocarp cells can increase hundreds of folds times from anthesis to mature stage (Cheniclet et al. 2005; Xiao et al. 2009). Generally, fruit size that is controlled by quantitative trait loci (QTLs) in tomato is largely defined by the number and volume of cells in the fruit, which are respectively attributed to cell division and cell expansion. Several genetic studies have identified a handful of QTLs responsible for the increases of fruit size or weight during tomato domestication and breeding improvement (Tanksley 2004). Two cloned fruit size QTLs in tomato impact fruit mass through the regulation of cell division. *fw2.2*, encoding a plant specific protein, negatively regulates cell division during early fruit growth, likely through its interaction with a regulatory subunit of the Casein Kinase II involving in cell cycle regulation (Frary et al. 2000; Cong and Tanksley 2006). *fw3.2* encoding a cytochrome P450 also affects cell number but not cell size in the fruit (Chakrabarti et al. 2013). Whereas, gain-of-function mutation in *fw11.3* allele (*fw11.3*-D) increases fruit weight cell size through its positive regulation of pericarp cell size (Mu et al. 2017). The enlargement of pericarp cells is associated with higher DNA ploidy in these cells, indicating that *fw11.3* may be involved in cell cycle regulation. Despite the progress in the identification of genetic loci controlling fruit size, the regulatory mechanism underlying cell division and expansion during tomato fruit development is still not well understood.

Endoreduplication as a specialized cell cycle in which bypasses mitosis is often observed in leaf, trichome and fruit where mass increase is rapid and metabolism is highly active (Inze and De Veylder 2006). During tomato fruit development, pericarp cells increase substantially from anthesis to maturation. Concomitantly, nuclear DNA content in these cells can reach as high as 512C (Cheniclet et al. 2005). Given there is a strong positive correlation between cell size and ploidy level, it has also been hypothesized that endoreduplication is likely one of the major driving forces to increase cell size in tomato fruit. However, DNA ploidy level is not always associated with cell size; high DNA ploidy due to enhanced endoreduplication may cause either no or subtle changes in cell size (De Veylder et al. 2001; Leiva-Neto et al. 2004).

Cell cycle in eukaryotes is governed by cyclin-dependent protein kinases (CDKs) and cyclins (CYC-CDKs) complexes, which are required for different target proteins involved in the transition between cell cycle phases (Inze and De Veylder 2006). Disruption of the CYC-CDK complex not only impairs mitosis but also endoreduplication. For example, loss-of-function mutants of the A-type cyclin genes *CYCA2;1* and *CYCA2;3* in Arabidopsis display an increase in DNA ploidy due to enhanced endoreduplication (Imai et al. 2006; Yoshizumi et al. 2006), and the triple mutant *cyca2;2/3/4* has fewer and much bigger leaf cells (Vanneste et al. 2011). Overexpression of a non-degradable *CYCB1* in tobacco cells enhances endomitosis (Weingartner et al. 2004). When overexpressing or silencing the D-type cyclin gene *CYCD5;1* in Arabidopsis, both the endoreduplication index (EI) and cell number in the leaf was affected (Sterken et al. 2012). Furthermore, the onset of endoreduplication is regulated by several important regulators including the CDK inhibitors KIP-RELATED PROTEIN/INTERACTOR OF CDKs (KRP)/ICK, the protein kinase WEE1, the degradation machinery for cell cycle proteins like anaphase promoting complex activator CCS52, and others (Chevalier et al. 2011; De Veylder et al. 2011; Fox and Duronio 2012; Heyman and De Veylder 2012; Chevalier et al. 2013; Hayashi et al. 2013; Jegu et al. 2013; Zielke et al. 2013). In tomato, through overexpression or antisense approaches, cell cycle components *SlCDKB1* and *SlCDKB2* have also been shown to regulate endoreduplication process, which overexpressing the two genes decreases EI and reduce fruit size (Czerednik et al. 2012). Whereas, repressing *SlCDKA1* expression does not change EI but caused cell number reduction (Czerednik et al. 2012). Down-regulation of *SlWEE1* in tomato also reduces EI and cell size and produces smaller fruits (Gonzalez et al. 2007). Intriguingly, both overexpressing and repressing *SlCCS52A* inhibit tomato fruit growth, causing dramatic reduction in fruit size (Mathieu-Rivet et al. 2010). These studies have demonstrated the core cell cycle components play important roles in the control of fruit size.

Zinc finger protein, including the C_2_H_2_ zinc finger proteins, is a large protein family. The C_2_H_2_ type zinc finger proteins can be classified into three sets -- A, B and C -- based on the number and arrangements of the fingers, for example, the members in set C contain either a single zinc finger or dispersed zinc fingers (Englbrecht et al. 2004). Set C can be further divided into C1, C2 and C3 subgroups according to the spacing between the two invariant histidine residues in the finger motif by three (C1), four (C2) or five (C3) amino acids, respectively. *SERRATE* (*SE*), *FERTILIZATION INDEPENDENT SEEDS 2* (*FIS2*), *VERNALIZATION 2* (*VRN2*) and *EMBRYONIC FLOWER 2* (*EMF2*) belonging to the C2 subgroup are involved in regulation of leaf morphology, flowering, flower development and seed formation (Chen et al. 1997; Luo et al. 1999; Gendall et al. 2001; Grigg et al. 2005). In this study, we characterized a C2-li member of C2H2 zinc finger gene *SlPZF1* that is highly expressed in pericarp of developing fruits and identified several regulators of cell cycle progression as SlPZF1 partners through yeast two hybrid screening. Our results demonstrate that SlPZF1 is a novel regulator of cell size during tomato fruit development, which its expression is crucial for fruit size control.

## Results

### *SlPZF1* was mainly expressed in pericarp during early fruit growth

**Fig. 1:**
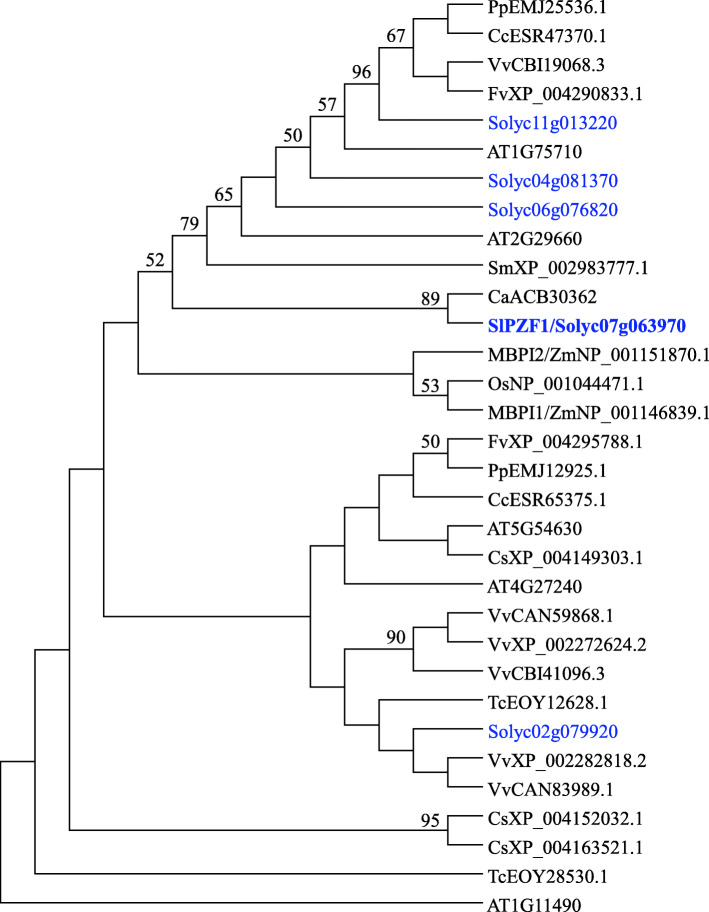
Phylogenetic tree analysis of SlPZF1 and its homologs from other plant species. The phylogenetic tree of SlPZF1 and its homologous proteins was constructed using the Maximum Parsimony method by MEGA5. The 50 amino acid sequences of the C_2_H_2_ motif from SlPZF1 and homologs from other species were used. Amino acid sequences for tomato (in blue) and Arabidopsis proteins were retrieved from Sol Genomics Network (http://solgenomics.net) and The Arabidopsis Information Resource (http://www.arabidopsis.org/), respectively. Other protein sequences were obtained from NCBI by blast search using SlPZF1 protein sequence. Proteins from plant species other than tomato and Arabidopsis were denoted by the first letters of their Latin names followed by accession number. Ca, *Capsicum annuum* (pepper); Cc, *Citrus clementina* (citrus); Cs, *Cumunis sativus* (cucumber); Fv, *Fragaria vesca* (strawbeery); Os, *Oryza sativa* (rice); Pp, *Prunus persica* (peach); Sm, *Selaginella moellendorffii* (club moss); Tc, *Theobroma cacao* (cocoa); Vv, *Vitis vinifera* (grape); Zm, *Zea mays* (maize)

We previously identified a set of putative transcription factors that were preferentially expressed in developing fruits (Xiao et al. 2009). Among them, *Solyc07g063970* encodes a zinc finger protein, sharing highest similarity in amino acid sequence with Arabidopsis protein AT5G54630 (61% identity), a C2-li zinc finger protein only presented in plants (Englbrecht et al. 2004). Blast search on public databases using Solyc07g063970 amino acid sequence as query, we identified additional four homologs in tomato genome. Using the 50 amino acid sequence of the conserved zinc finger motif predicted by MEME (Bailey et al. 2009), we constructed Maximum Parsimony tree for Solyc07g063970 and C2-li members from other plant species by MEGA5 (Tamura et al. 2011) (Fig. 1). The phylogenetic tree placed Solyc07g063970 and other three tomato homologs in the subgroup containing the maize ZmMBPI-1 and ZmMBPI-2, which have been shown to interact with one repeat MYB transcriptional factor ZmMRP-1 involving in the regulation of transfer cell layer formation in maize (Gomez et al. 2002; Royo et al. 2009).

**Fig. 2:**
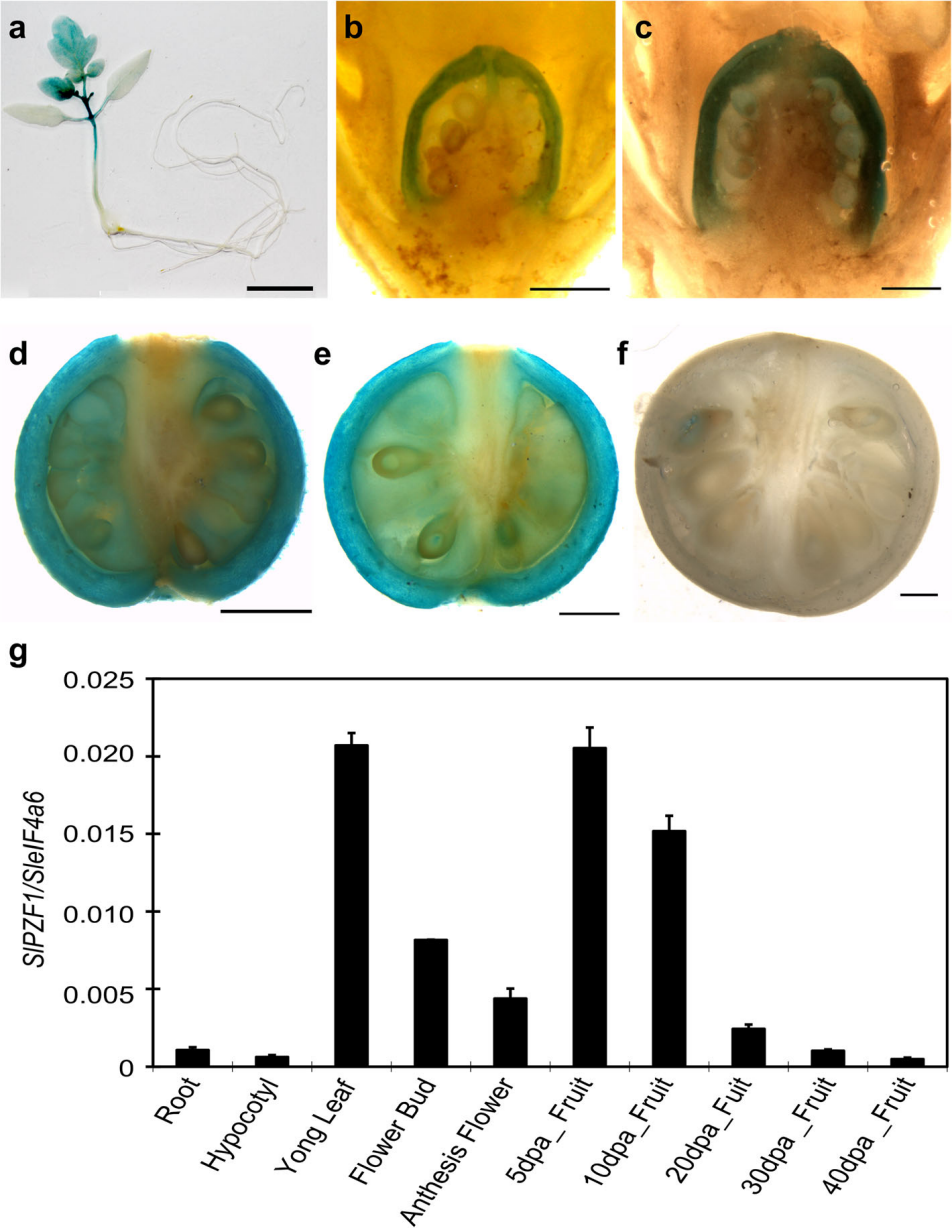
Expression pattern of the *SlPZF1* gene. **a**, GUS expression in a representative *pSlPZF1::GUS* seedling of plants. Bar = 1 cm. **b-f**, GUS expression in young flower buds (**b**), anthesis flowers (**c**), and in fruits at 5 (**d**), 10 (**e**) and 30 (**f**) DPA. Flowers and fruits were cut longitudinally before staining. **g**, Quantitative reverse transcription PCR (qRT-PCR) analysis of *SlPZF1* expression in vegetative tissues, flowers and fruits at different stages. Roots, stems and leaves were collected from 7 DAG (days after germination) seedlings of LA1781. YFB, young flower buds; AnFl, anthesis flowers. *n* = 3. Data represents mean ± SD. Bar = 1 mm (**b-e**) and 2 mm (**f**), respectively

To confirm its expression in developing fruits, we investigated the spatial-temporal expression pattern of *Solyc07g063970* using its native promoter-driven GUS reporter lines and qRT-PCR. The GUS expression under the 2.3 kb promoter of *Solyc07g063970* was observed in young leaves, flowers and developing fruits, but not in roots (Fig. 2a). During flower and fruit development, GUS expression was specifically detected in ovary pericarp of unopened and anthesis flowers, and then in fruit pericarp at 5 and 10 DPA (days post anthesis, Fig. 2b-e). GUS expression was undetectable in 30 DPA fruits (Fig. 2f). Quantification of expression levels by qRT-PCR further confirmed that *Solyc07g063970* was mainly expressed in young leaves, flowers, and young fruits at 5 and 10 DPA (Fig. 2g). Given its specific expression in the pericarp of ovaries and developing fruits, we named *Solyc07g063970* as *SlPZF1* (*Solanum* *lycopersicum* *PERICARP-associated* *ZINC* *FINGER* *PROTEIN* *1*).

### Altered *SlPZF1* expression affects fruit growth by its action on cell size

**Fig. 3:**
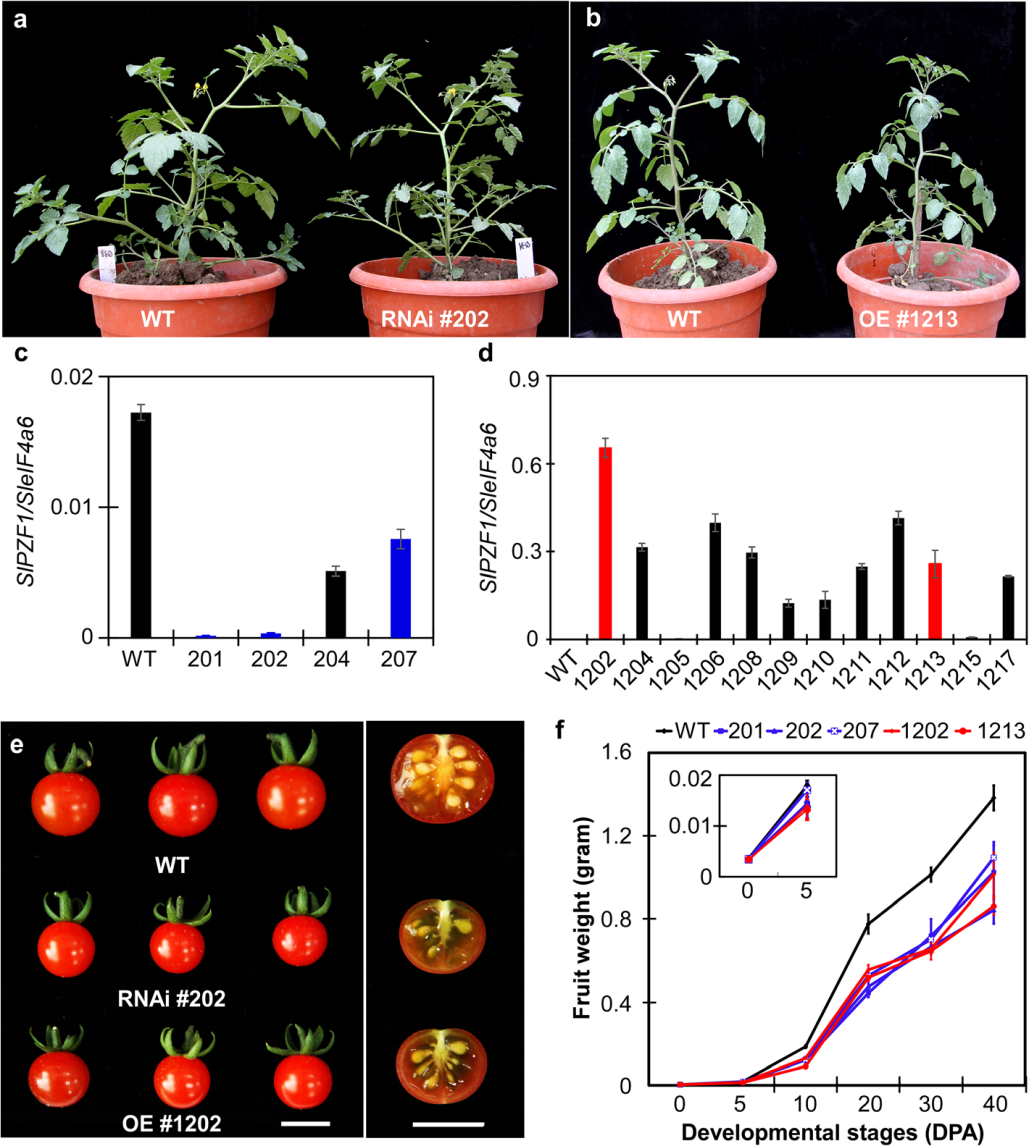
Fruit phenotypes caused by altered *SlPZF1* expression. **a-b**, representative plants of *SlPZF1* RNAi (**a**) and OE (**b**) lines. **c**, expression levels of *SlPZF1* in four RNAi lines, compared to the wild type LA1781. The *SleIF4a6* was used as loading control (reference) to calculate the relative expression levels of the *SlPZF1* gene. *n* = 3. **d**, expression levels of *SlPZF1* in 12 OE lines. *n* = 3. **e**, Representative ripe fruits of *SlPZF1* OE, RNAi and wild type. On the right side, cross-sectioned fruits were shown. Bar = 1 cm. **f**, fruit growth rate in two *SlPZF1* RNAi and three OE lines, compared to the wild type. *n* = 30–50

To better understand its roles in fruit growth, we generated tomato transgenic plants in *S. pimpinellifolium* LA1781 background either overexpressing *SlPZF1* under the 35S promoter (OE lines) or repressing its expression via RNA interference (RNAi lines). We obtained 20 and 7 independent OE and RNAi lines, respectively. The OE and RNAi plants displayed no visible phenotypic abnormity in plant stature and flowering time, compared to their non-transgenic siblings (wild type) (Fig. 3a, b). We for further analysis selected two respective independent OE and three RNAi lines, which showed dramatically increased (OE lines) or decreased expression of the *SlPZF1* gene (Fig. 3c, d). Smaller fruits were first noticed for these *SlPZF1* OE and RNAi lines (Fig. 3e). To further investigate the effect of the altered *SlPZF1* expression on fruit growth, we compared fruit growth rates of the five selected independent lines with the wild type. To minimize the impact of any mutations introduced by tissue culture, these lines were backcrossed more than three times to the wild type LA1781 plants and phenotypic measurements were done on homozygous plants. The results showed that the two OE and three RNAi lines produced smaller fruits compared to wild type, respectively, and the differences were apparent starting from 10 DPA (Fig. 3f). This suggests that *SlPZF1* regulates early fruit growth.

**Fig. 4:**
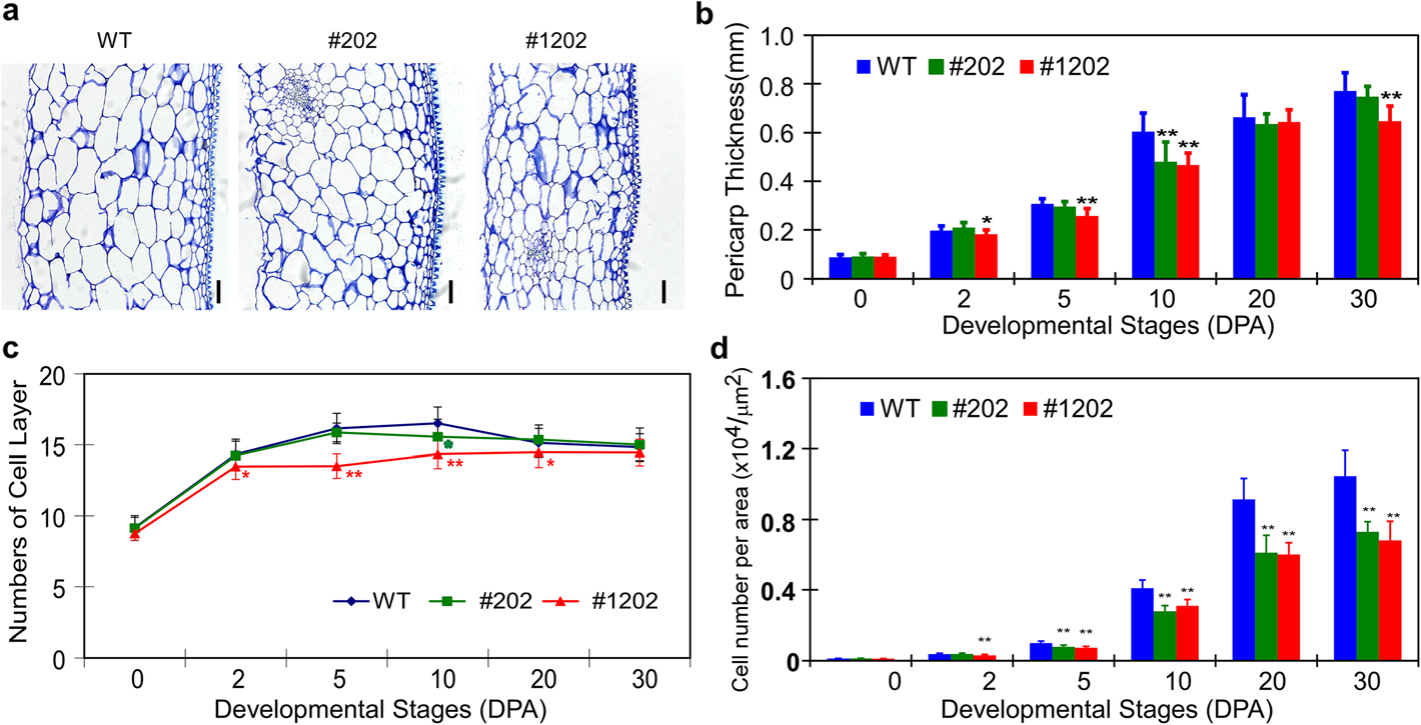
*SlPZF1* regulates pericarp width and cell size. **a**, representative pericarp sections of the *SlPZF1* OE (line #1202), RNAi (#202) and wild type (WT) fruits. Bar = 100 μm. **b**, pericarp thickness of the *SlPZF1* OE (line #1202), RNAi (#202) and wild type (WT) fruits. **c**, the number of cell layers of the pericarp tissues from the *SlPZF1* OE (line #1202), RNAi (#202) and wild type (WT) fruits. **d**, cell numbers per area in the mesocarp regions of the *SlPZF1* OE, RNAi and wild type fruits. The averaged pericarp thickness, the number of cell layers from epidermis to endodermis, and the number of mesocarp cell number per area were based on at least 10 fruits for each line. Statistical significance was based on Student’s t-test. *, *p* < 0.05; **, *p* < 0.01. Data represents mean ± SD

Because *SlPZF1* was mainly expressed in the pericarp during fruit development, we compared the cell morphology between the two representative lines (OE line #202) and (RNAi line #1202) to wild type. At 30 DPA when the fruits were at breaker stage and reached to the final size, the pericarp of the OE fruits contained smaller cells and looked thinner compared to wild type (Fig. 4a). The RNAi fruits also contained smaller pericarp cells but the pericarp thickness was comparable to wild type. We then performed a time course analysis of pericarp growth from anthesis to 30 DPA by measuring the pericarp thickness, the number of cell layers from epidermis to endodermis that marks the cell division activity, and the mesocarp cell sizes that were deduced from counted cell number per area. The results showed that overexpressing *SlPZF1* caused a significant reduction in the thickness and the mesocarp cell size of the pericarp from 2 to 30 DPA, while the number of pericarp cell layers at 30 DPA was not affected though was reduced earlier (Fig. 4b-c). In contrast, no substantial difference in pericarp thickness and the number of cell layers was observed in the RNAi fruits except at 10 DPA, but smaller pericarp cells were noticed since 5 DPA (Fig. 4d). At 30 DPA, there was no difference in the number of pericarp cell layers among OE, RNAi and wild type fruits, indicating that *SlPZF1* mainly regulates cell size, at least in the pericarp. Thus, the results suggest that *SlPZF1* controls fruit size through its action on cell size.

### Cell size reduction by altered *SlPZF1* expression is associated with attenuated endoreduplication

**Fig. 5:**
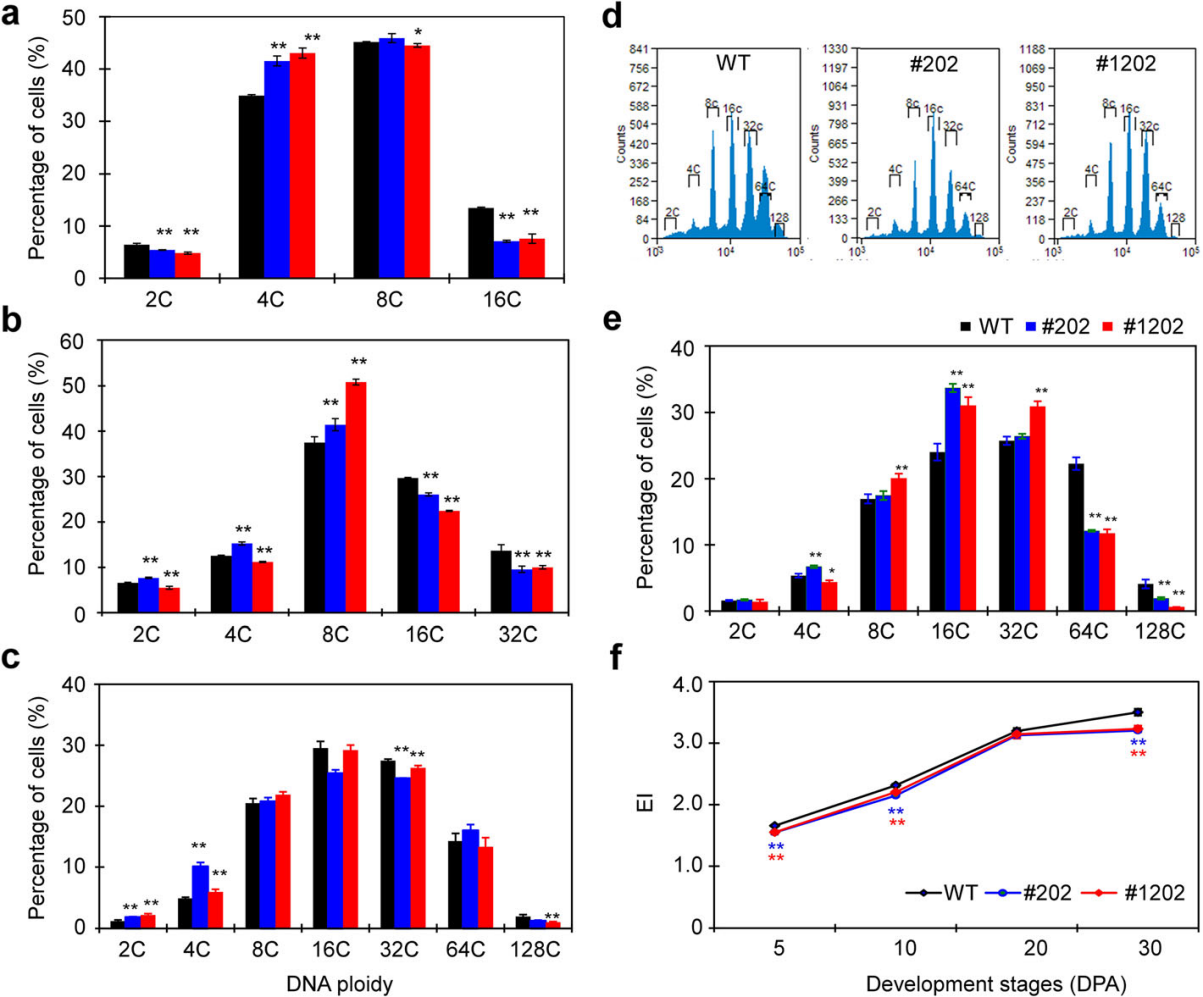
Ploidy level distribution in the pericarp of the *SlPZF1* OE and RNAi fruits. **a-e**, distributions of pericarp cells with different DNA ploidy levels at 5 (**a**), 10 (**b**), 20 (**c**) and 30 (**d, e**) DPA. Representative flow cytometry profiles of the nuclei of pericarp cells at 30 DPA was shown in (**d**). **f**, dynamic changes in endoreduplication index (EI) of the pericarp cells of the *SlPZF1* OE, RNAi, and wild type fruits from 5 to 30 DPA. For each line, pericarp tissues from at least three fruits per plant were subjected to flow cytometric analysis, and 8000–15,000 cells were counted for each sample. Data represents mean ± SD

Shift from mitosis to cell expansion often occurs within one-week post pollination in small-fruited *S. piminellifolium* accessions (Xiao et al. 2009). In tomato, pericarp cell size is positively correlated to DNA ploidy level (Cheniclet et al. 2005). Given altered *SlZFP1* expression caused reduction in pericarp cell size, we reason that *SlPZF1* may play a role in regulation of the transition from mitotic cell cycle to endoduplication cycle during fruit growth. Using flow cytometry, we monitored the changes in DNA ploidy level of pericarp cells of transgenic lines #202 and #1202 from early cell expansion (5 DPA) to breaker stage (30 DPA). At 5 DPA, the maximum DNA ploidy level of pericarp cells was 16C. Comparing to wild type, the proportions of16C cells were much fewer in the OE line #1202 and RNAi line #202 (Fig. 5a). Later, the proportions of pericarp cells with maximal ploidy DNA levels were still lower in the OE and RNAi fruits, despite cell proportions of particular DNA ploidy levels had considerable differences between the OE and the RNAi lines (Fig. 5b-e).

Then, endoreduplication indices (EIs) were calculated based on the weighted percentages of nuclei with different DNA contents (Barow and Meister 2003). The EIs of WT pericarp increased from about 1.7 at 5 DPA to 3.5 at 30 DPA, whereas the pericarp cells of OE and RNAi lines had much lower EIs at 5 DPA and afterward (Fig. 5f), indicating that endoreduplication was weakened in the fruits of OE and RNAi lines.

### Overexpression of *SlPZF1* affects expression of a subset of cell cycle regulators

**Fig. 6:**
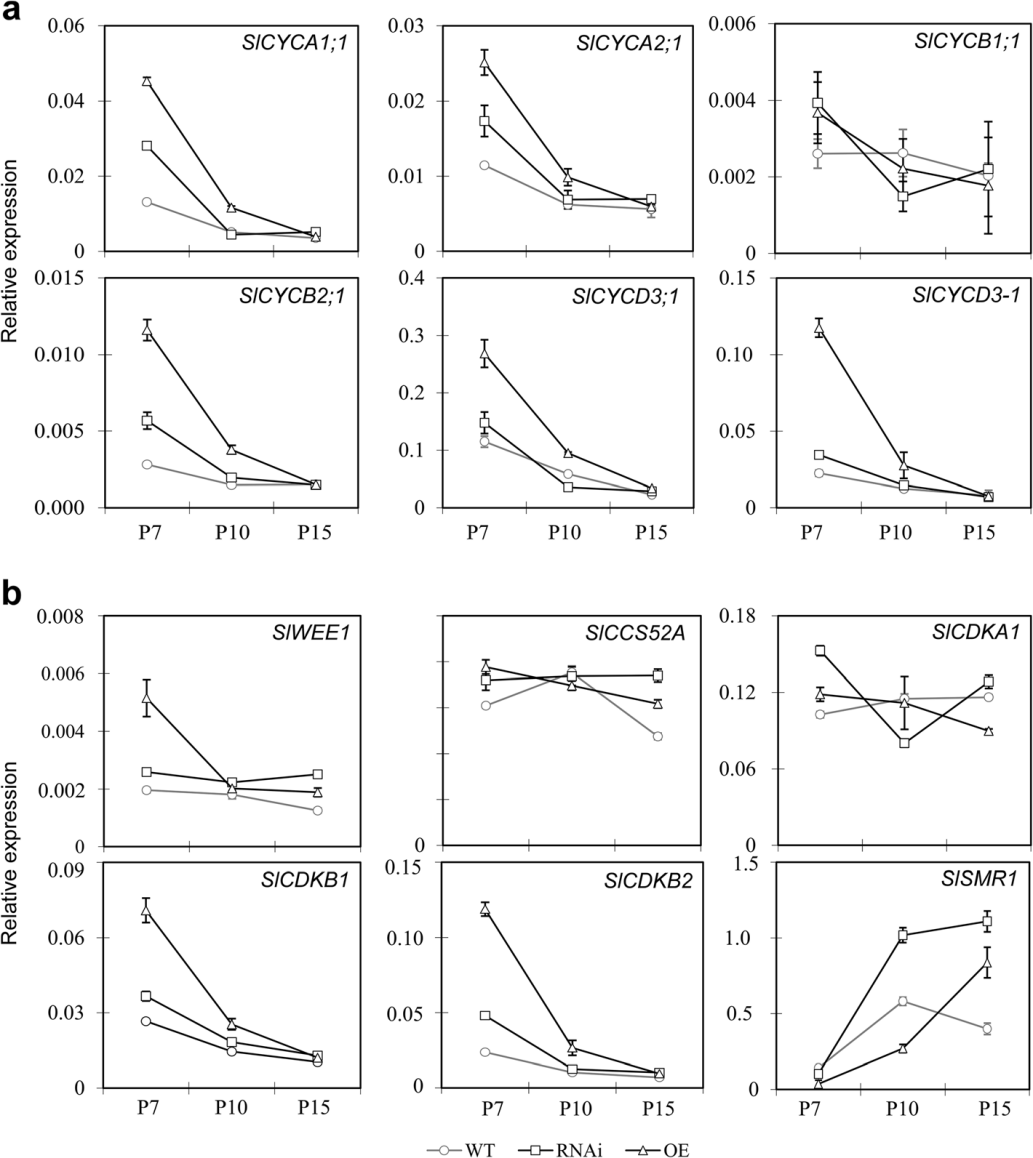
Expression changes of cell cycle regulators in the pericarp by altered *SlPZF1* expression. **a**, expression of cyclin genes in the pericarp of the *SlPZF1* OE, RNAi and wild type fruits at 7, 10 and 15 DPA. **b**, expression of *SlCCS52A*, CDKs and CDK inhibitors in the pericarp of the *SlPZF1* OE, RNAi and wild type fruits at 7, 10 and 15 DPA. The qRT-PCR was conducted on total RNA extracted from pooled samples from three plants at the same growth stages. Expression level was normalized to *SleIF4a6* and data are means ± sd. *n* = 3

Transition from mitotic cell cycle to endoreduplication cycle requires precise regulation of the CYC-CDK activity (Inze and De Veylder 2006; Fox and Duronio 2012; Chevalier et al. 2013). Because the difference in cell size and pericarp thickness was observed as early as 10 DPA in the fruits of *SlPZF1* OE and RNAi lines (Fig. 4b, d), to test whether *SlPZF1* regulates cell size through modulating gene expression involved in cell cycle regulation, we performed quantitative reverse transcribed PCR (qRT-PCR) analysis of genes known for their roles in cell cycle regulation in fruit pericarp at cell expansion stage (7–15 DPA). In tomato, the cyclin genes, *SlCYCA1;1*, *SlCYCA2;1, SlCYCB1;1*, *SlCYCB2;1*, and *SlCYCD3;1*, are expressed at relatively high levels in the pericarp of young fruits (Joubes et al. 2000). Except *SlCYCB1;1*, expression of the rest four cyclin genes and another highly expressed D-type cyclin gene *SlCYCD3–1* in the 7 and 10 DPA pericarp were elevated by overexpression of *SlPZF1*, but not by RNAi repression; their expression was slightly or not elevated in the pericarp of developing RNAi fruits (Fig. 6a). Further expression analysis of these cyclin genes in whole flowers and fruits at early development stages revealed that only *SlCYCA2;1* expression was substantially increased and slightly repressed in the flowers and young fruits of the overexpression and RNAi lines, respectively (Supplementary Fig. S1).

We then further checked the expression of cell cycle regulators *SlCDKA1*, *SlCDKB1*, *SlCDKB2*, *SlWEE1*, *SlCCS52A* and *SlSMR1* (*SIAMESE-RELATED 1*). Similarly, overexpression of *SlPZF1* had stronger effects on the expression of these regulators in pericarp; *SlWEE1*, *SlCDKB1* and *SlCDKB2* were up-regulated in the pericarp of OE but not RNAi fruits at 7 DPA (Fig. 6b). Expression of *SlCDKA1* and *SlCCS52A* was not impacted by overexpression or suppression of the *SlPZF1* gene. The SIAMESE-RELATED (SMR) family members of plant specific CDK inhibitors regulate the transition from the mitotic cell cycle to endoreduplication (Kumar et al. 2015). In wild type pericarp at early cell expansion stage, *SlSMR1* expression peaked around 10 DPA. At this stage, *SlSMR1* expression was respectively decreased and increased *SlPZF1* in the pericarp of OE and RNAi fruits (Fig. 6b). *SlSMR1* expression was maintained at high levels at 15 DPA when its expression in wild type had decreased.

### SlPZF1 interacts with proteins involved in cell cycle progression

**Fig. 7:**
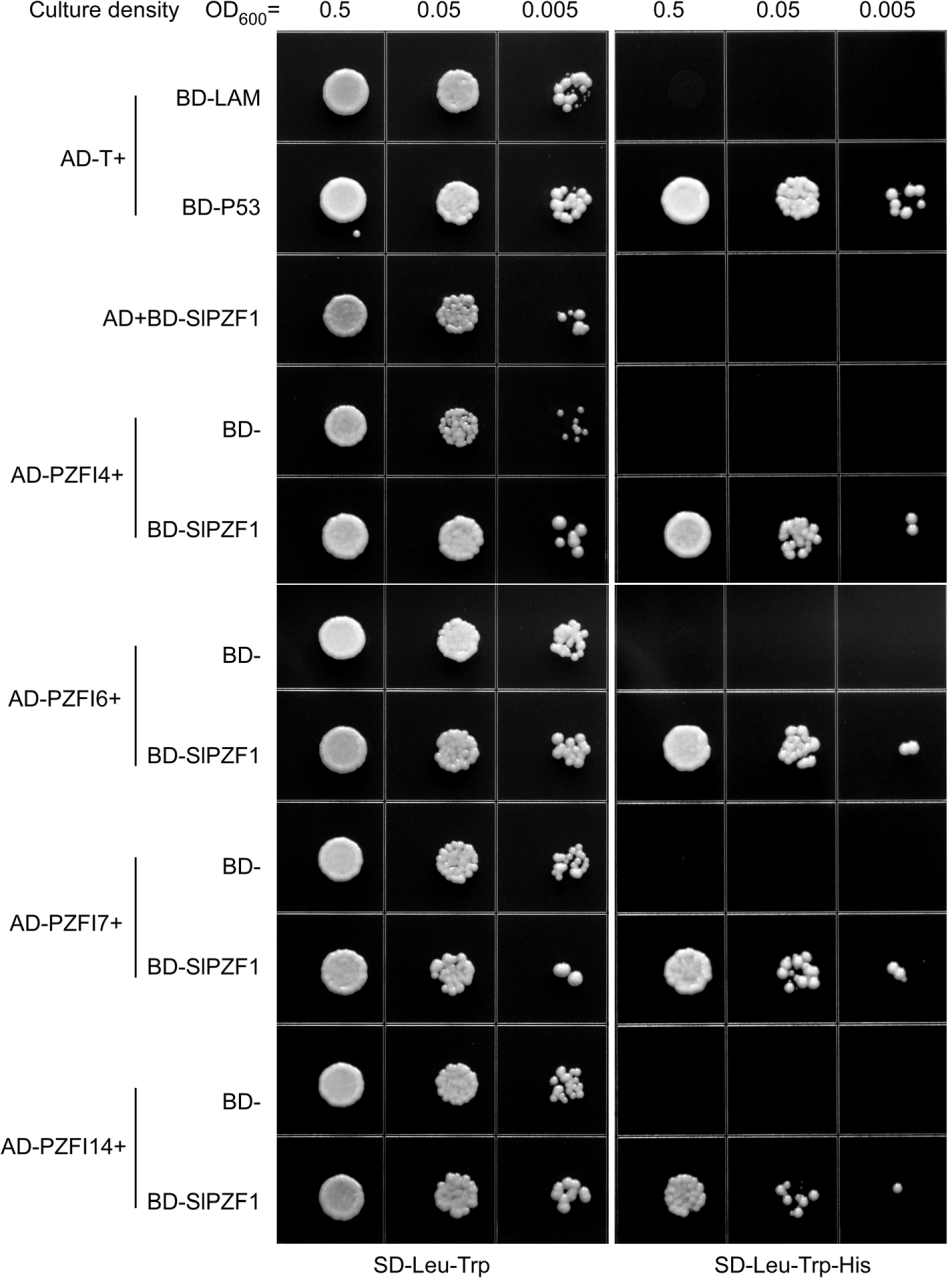
SlPZF1 interacted with unconventional regulators of cell cycle progression in yeast cells

To gain insight in the biochemical functions of SlPZF1 in fruit development, we performed a yeast two hybrid screen to identify proteins interacting with SlPZF1 (PZFIs). The cDNA library we screened was made from Heinz1706 fruits at different stages (from anthesis to ripen). By screen around 1 million clones, we identified 14 different proteins (PZFI1–14) that interacted with SlPZF1 in yeast cells (Table 1 and Fig. 7). Among them, PZFI4, 6 and 14 are likely involved in cell cycle progression. PZFI4 is a pre-mRNA-splicing factor, which its Arabidopsis homolog SWELLMAP 1 regulates the timing of cell cycle arrest during leaf development (Clay and Nelson 2005). PZFI6 is PAPA-1-like conserved region family protein, a nucleolar protein that can induce cell cycle arrests at the G1 phase in mammal cells (Kuroda et al. 2004). PZFI14 is basic helix-loop-helix (bHLH) protein, and its closest homolog of Arabidopsis LONESOME HIGHWAY (LHW) is a key regulator of vascular cell divisions (Vera-Sirera et al. 2015). PZFIs also include four putative regulators of genomic integrity in mitotic cells, for example, a Phox domain-containing protein (PZFI7), Microspherule protein 1 (PZFI1), and two Fanconi anemia complex subunits (PZFI3 and PZFI11). In addition, *PZFI2*, encoding a cysteine-rich extensin-like protein, was co-expressed with *SlPZF1* based on correlation of gene expression during tomato fruit development (Tomato Expression Atlas, http://tea.solgenomics.net). Both *PZFI2* and *SlPZF1* were highly expressed in the pericarp of early developing fruits (Supplementary Fig. 2a, b). Gene ontology analysis of the 1137 genes co-expressed with *SlPZF1* revealed that the most enriched Go-slim term was cell cycle in biological processes and DNA replication in pathways (Supplementary Fig. 2c). These results imply that *SlPZF1* is involved in cell cycle regulation.

**Table 1:** SlPZF1-interacting proteins identified by Yeast Two Hybrid

PZFI	ITAG name	No of clones	Description
PZFI1	Solyc07g032510	9	Microspherule protein 1
PZFI2	Solyc01g006400	4	Cysteine-rich extensin-like protein-4
PZFI3	Solyc03g043870	2	Ubiquitin ligase protein FANCL
PZFI4	Solyc09g072570	2	Pre-mRNA-splicing factor SLU7-A; SWELLMAP 1/SlSMP
PZFI5	Solyc11g066060	2	Heat shock protein 70
PZFI6	Solyc01g079350	1	PAPA-1-like conserved region family protein
PZFI7	Solyc03g116830	1	Phox domain-containing protein
PZFI8	Solyc04g077740	1	Unknown Protein
PZFI9	Solyc06g068130	1	TPR domain protein
PZFI10	Solyc07g062970	1	protein phosphatase 2C dig3
PZFI11	Solyc08g014020	1	MLP3.2 protein
PZFI12	Solyc08g079250	1	Plant lipid transfer protein
PZFI13	Solyc10g047290	1	Protein phosphatase 2C
PZFI14	Solyc11g068960	1	Basic helix-loop-helix dimerisation region bHLH; LONESOME HIGHWAY/SlLHW

**Fig. 8:**
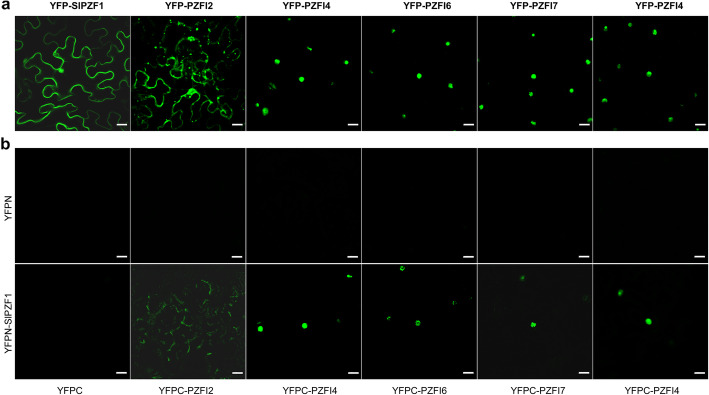
SlPZF1 interacted with PZFIs in *N. benthamiana* leaves. **a**, subcellular localization of SlPZF1 and five PZFIs. **b**, BiFC verification of the interactions between SlPZF1 and five PZFIs in *N.benthamiana* leaves. YFP signals were monitored after 2 days post infiltration under a confocal laser scanning microscope. YFPN, N-terminal part of YFP; YFPC, C-terminal part of YFP. Bar = 20 μm

We further verified the interactions between SlPZF1 and five PZFIs that are putatively involved in cell expansion and cell cycle progression by BiFC in *N. benthamiana* leaves. First, we investigated the subcellular localization of SlPZF1 and five PZFIs (PZFI2, 4, 6, 7 and 14) fused with the fluorescence protein YFP by transient assays in *N. benthamiana* leaves. YFP-SlPZF1 was located to the nucleus and cytosol, where YFP-PZFI4, YFP-PZFI6, YFP-PZFI7 and YFP-PZFI14 were located to the nucleus (Fig. 8a). YFP-PZFI2 was located to the cytosol and microbodies. The BiFC assay further confirmed the interactions between SlPZF1 and the five selected PZFIs in vivo (Fig. 8b; Supplementary Fig. S3).

## Discussion

Genetic analyses have identified a number of QTL loci controlling tomato fruit size, which is largely defined by cell number and cell size (Tanksley 2004). The cloned QTL loci *fw2.2*, *fw3.2*, *fas* and *lc* are involved in regulation of cell number either by acting on the formation of primary cell population before pollination or mitotic activity after fruit set (Frary et al. 2000; Munos et al. 2011; Chakrabarti et al. 2013; Xu et al. 2015; Chu et al. 2019), while *fw11.3* functions as a cell size regulator to control fruit size (Mu et al. 2017). *FW11.3* increases cell size predominantly in fruit pericarp, likely by enhancing endoreduplication. Endoreduplication is important for cell expansion during tomato fruit development based on altered cell morphology by disturbed expression of genes regulating the exit of cell cycle (Cheniclet et al. 2005; Chevalier et al. 2011; Chevalier et al. 2013). In this study, we demonstrated that the zinc finger gene *SlPZF1,* which was mainly expressed in early developing pericarp of tomato fruits, is a new cell size regulator through its action on endoreduplication.

### *SlPZF1* as a potential pericarp marker gene

Cell division and expansion in tomato fruit is rapid during early fruit growth as it has been shown that mitosis completes within 1 week in the small fruited species *S. pimpinellifolium* followed by drastic cell expansion for 2 to 3 weeks (Xiao et al. 2009). Two cloned fruit size QTL loci, *fw2.2* and *fw3.2*, regulate cell division during tomato fruit growth. *fw2.2* likely acts through casein kinase CKII to regulate mitotic cell cycle (Frary et al. 2000; Cong and Tanksley 2006), whereas *fw3.2/SlKLUH* encoding a cytochrome P450 enzyme regulates cell number though the molecular mechanism underlying remains to be revealed (Chakrabarti et al. 2013), likely by regulating cell proliferation duration as its Arabidopsis homolog does (Anastasiou et al. 2007). *fw3.2/SlKLUH* also regulates side shoot growth and ripening time, suggesting it has broad actions in plant development. The two genes are expressed in early growing fruits but exact expression domains remain unknown.

The growth of pericarp has been coordinated with other fruit parts to ensure proper development of the fruit as a whole. Thus, the mitotic activity in pericarp is often used as a reliable indicator of fruit growth. Identification of pericarp-specific genes will provide valuable molecular markers for dissection of cell proliferation and cell growth in this fruit tissue. Despite it mainly regulates the enlargement of pericarp, *FW11.3/CSR* is expressed at relatively higher levels in the columella rather than in the pericarp (Mu et al. 2017). In contrast, GUS expression driven by *SlPZF1* promoter was detected specifically in the pericarp during flower and fruit development, very low if not absent, in other parts of the flower or the fruit. Although *SlPZF1* was also expressed in young leaves and hypocotyls, it may not play important roles in leaf development since no obvious phenotypic change in leaf development was observed in the *SlPZF1* OE and RNAi lines. Therefore, *SlPZF1* may be used as pericarp marker for analyzing pericarp-associated cell activity. *SlPZF1* as a pericarp-associated gene is also supported by its functionality in regulation of pericarp cell size.

### Cell size regulation by *SlPZF1*-mediated endoreduplication

Endoreduplication is hypothesized as one of the major factors to determine fruit size in tomato (Cheniclet et al. 2005; Chevalier et al. 2011; Chevalier et al. 2013). The evidences to support the hypothesis mainly come from functional analysis of conserved cell cycle genes. It is well established that the transition from mitotic cell cycle to endoreduplication is controlled by the CYC-CDK activity, which are regulated at transcriptional and post-translational levels (Inze and De Veylder 2006; Inagaki and Umeda 2011). For example, enhanced endoreduplication (higher EI) in tomato pericarp cells can be achieved by repressing *SlCCS52B* expression (Chevalier et al. 2013), whereas lower EIs have been obtained by overexpressing one of the two cyclin dependent kinases *SlCDKB1* and *SlCDKB2* or repressing the expression of *SlCCS52A* or *SlWEE1* (Gonzalez et al. 2007; Mathieu-Rivet et al. 2010; Czerednik et al. 2012). In these transgenic lines, fruit size is positively correlated to EI. Similarly, the *SlPZF1* transgenic fruits contained smaller pericarp cells associated with lower EIs, and the EI’s kinetic changes were also well correlated to fruit mass changes during fruit growth, implying that endoreduplication contributes substantially to fruit growth in tomato.

Generally, inhibiting cell division often triggers enhanced cell expansion, suggesting that compensatory mechanisms are involved in maintaining the final size of plant organs (Hisanaga et al. 2015). For example, Arabidopsis plants overexpressing the cyclin-dependent kinase inhibitor *Kip-related protein 2* (*KRP2*) have fewer but larger leaf cells (De Veylder et al. 2001). However, several studies indicate that compensatory mechanisms may not always come into play. Overexpressing *AINTEGUMENATA* (*ANT*) increases cell number but cell size remains unchanged (Mizukami and Fischer 2000). Similar phenomenon has been also observed in tomato fruits overexpressing *SlCCS52A*, which overexpression does not affect the number of pericarp cell layers but causes reduction in cell size due to early inhibited endoreduplication (Mathieu-Rivet et al. 2010). In the case of *SlPZF1*, overexpression inhibits cell division and the effect is not compensated by cell expansion, causing defect in endoreduplication. These observations suggest that endoreduplication-mediated cell growth in tomato fruits may be uncoupled from cell division.

### Possible roles of SlPZF1 in regulation of fruit growth

Several examples show that similar phenotypes can be caused by overexpression and down-regulation of a particular gene. For example, both *SlCCS52A*-overexpressing and antisense lines produce smaller fruits containing smaller pericarp cells, despite the delayed endoreduplication in early growing fruits is resumed later (Mathieu-Rivet et al. 2010). *CCS52A* also has similar effects on leaf development in Arabidopsis (Liu et al. 2012; Baloban et al. 2013). The overexpression plants of *SMP1* or *RGB1* -- encoding the heterotrimeric G protein subunit β -- show similar phenotypes with their loss-of-function mutants or RNAi plants in leaf and stem development (Clay and Nelson 2005; Sun et al. 2014). In addition, altering the expression of *formation of haploid and binucleate cells1* (*FAB1A/B*), which encodes a phosphatidylinositol 3-phosphate 5-kinase, by RNAi and overexpression, impairs vacuolar acidification and endocytosis in Arabidopsis (Hirano et al. 2011). We speculate that for these genes balanced expression is critical for their biochemical activities.

Overexpressing *SlPZF1* elevated the expression of several cyclin genes and two CDKs (*SlCDKB1* and *SlCDKB2*) in the pericarp at 7 DPA, suggesting that overexpressing *SlPZF1* likely delays the transition of mitotic cell cycle to endoreduplication. Since overexpression of *CYCD3;1* in Arabidopsis (Dewitte et al. 2003; Dewitte et al. 2007), *SlCDKB1* and *SlCDKB2* in tomato (Czerednik et al. 2012) weakens endoreduplication, elevated expression of the three gene at early stage is likely responsible for the reduction in cell size and DNA ploidy in *SlPZF1-*overexpressing fruits.

The role of SlPZF1 in cell cycle regulation is further supported by its interactions with several proteins putatively involved in cell cycle progression and cell growth. Among them, PZFI4 is a putative pre-mRNA-splicing factor, sharing similarity with step II splicing factors. Its Arabidopsis homolog *SMP1* is hypothesized to control the timing of cell cycle arrest during leaf development (Clay and Nelson 2005). Thus, it is possible that *SlPZF1* together with *PZFI4* regulates cell cycle duration. Interestingly, *PZFI2*, encoding an extensin-like protein was co-expressed with *SlPZF1* during fruit development, and this extension-like gene been thought to play a role in the control of cell wall extensibility (Van den Heuvel et al. 2002). In plant, cell growth and cell cycle progression must be balanced for organ growth, which requires coordinated regulation of cytoplasmic growth, cell-wall extension, mitotic cell cycle, and endoreduplication (Thompson 2005; Sablowski and Carnier Dornelas 2014; Sablowski 2016). The interactions of SlPZF1 with the extension-like protein PZFI2 and cell cycle regulators including PZFI4 suggest that SlPZF1 may coordinate the interplay between cell wall extension and cell cycle during fruit growth.

Since no or very subtle change in the expression of cell cycle regulators was detected in the pericarp of *SlPZF1* RNAi fruits, SlPZF1 likely does not directly regulate transcription. However, we can’t rule out the possibility that SlPZF1 is involved in transcriptional regulation of some cell cycle regulators given several PZFIs including the bHLH transcription factor SlLHW are nuclear localized proteins. Though the functions of SlLHW in tomato fruit development are not known, *LHW* is mainly expressed in pericycle-vascular mother cells to regulate cell division, but not cell growth in *Arabidopsis* (Ohashi-Ito and Bergmann 2007; Ohashi-Ito et al. 2013; Smet et al. 2019). Due to very limited genetic and molecular information available for the identified SlPZF1-interacting proteins, it still needs to further explore the molecular mechanism underlying the regulation of cell cycle by SlPZF1 and its interacting partners during fruit development. Nonetheless, we demonstrate that *SlPZF1* plays an important role in the control of fruit size in tomato.

## Conclusion

*SlPZF1* encodes a member of C2H2 zinc finger protein family, preferentially expressed in the pericarp during tomato fruit development. Functional analysis reveals that *SlPZF1* interacting with several cell cycle regulators controls fruit size through its action on cell size regulation. SlPZF1 not only plat an important role in controlling fruit growth but also can be used as a pericarp marker to dissect the molecular mechanism underlying cell size regulation mediated by cell cycle in freshly fruits.

## Methods

### Plant materials and growth conditions

Seeds of *S. pimpinellifolium* LA1781 were obtained from the Tomato Genetics Resource Center at University of California, USA (http://tgrc.ucdavis.edu/). All plants including transgenic lines were grown in a phytotron at 20–25 °C under the condition of 70–80% humidity, and illuminated for 16 h daily by 150 mE·m^− 2^·s^− 1^ light from metal halide and high-pressure sodium lamps. Plants were fertilized weekly with all-purpose fertilizer and watered as needed.

### Generation of transgenic lines

The *SlPZF1* coding sequence was amplified from cDNA of LA1589 by RT-PCR using the primer pair XP0687 and XP0688, a Kozak sequence was added right ahead of the start codon ATG of *SlPZF1* (primer information used in this study can be found Supplementary Table S1). The two primers also contain restriction enzyme sites of *XbaI* and *SacI* to facilitate subsequent cloning, respectively. PCR product was cloned into the pGEM T-easy vector (Promega Beijing, Beijing, China) and verified by sequencing. To overexpress *SlPZF1*, the *p35S::SlPZF1* construct was made by placing the full length *SlPZF1* cDNA released by *XbaI* and *SacI* digestion in between the *CaMV 35S* promoter and the *NOS* terminator of the binary vector pHX20 derived from pZH01 digested by *XbaI* and *SacI* (Xiao et al. 2003). For construction of the *SlPZF1* RNAi vector, the 395 bp fragments containing *SlPZF1* 5′-UTR and partial coding sequence (− 78 to 317 bp) was amplified using the primers XP0791 and XP0792, followed by cloning into the binary vector pFGC5941 in both the sense and antisense directions, respectively (Kerschen et al. 2004). To make the GUS reporter driven by native *SlPZF1* promoter, a 2.3 kb fragment upstream of its coding sequence amplified from genomic DNA of wild type tomato using primers XP1027 and XP1073 was placed at the upstream of the *GUS* coding sequence, then the expression cassette was cloned into the binary vector pCIB10G (Xiao et al. 2008).

*Agrobacterium tumefaciens* strain GV3101 harboring the three respective plasmids were used for plant transformation using LA1781 cotyledons as explants according to the method previously described (McCormick 1991).

### Phenotypic measurement of transgenic lines

Genotyping of transgenic lines were conducted by PCR using primer pairs XP0515/XP0516 (*HygR* gene) and XP0517/XP0518 (*BarR* gene) for selecting *SlPZF1* OE and RNAi plants, respectively. To minimize the impact of somatic variations introduced during tissue culture on fruit traits measured, phenotypic analysis was conducted on the progenies of OE and RNAi lines that were backcrossed at least three times with LA1781 and their non-transgenic siblings were used as wild type controls.

For pericarp morphology, fruits at 0, 2, 5, 10, 20 and 30 DPA were fixed in FAA solution and embedded in Paraplast Plus (Sigma, USA). For each genotype, transverse sections of five fruits per timepoint were made at the middle of the fruit and briefly stained with 0.04% (W/V) toluidine blue solution. Then, the stained pericarp was observed under a dissection microscope (Leica M125/DFC 420, Germany). Pericarp thickness, the number of cell layers from epidermis to endodermis and mesocarp cell size was measured on images token from parafilm sections using the ImageJ software (http://rsbweb.nih.gov/ij/). For cell size measurements, mesocarp cells in defined areas were counted.

### Real time quantitative RT-PCR (qRT-PCR)

Total RNA was extracted from tomato tissues using Trizol reagent (Invitrogen, USA) based on the methods previously described (Xiao et al. 2009). For fruit tissues, either only pericarps at 7, 10 and 15 DPA (presented in Fig. 6) or whole flowers/fruits (presented in Supplementary Fig. S1) were collected for RNA extraction. Residual genomic DNA in the RNA samples was removed by RNase-free DNase according to protocol of RNase-free DNase I set (QIAGEN, Germany) and RNeasy® MinElute™ Cleanup kit (QIAGEN, Germany). Five microgram of DNase-treated total RNA was used to synthesize first strand cDNA using the First Strand cDNA Synthesis Kit (Thermo Fisher Scientific (China), China) and qRT-PCR was performed on three biological replicates using SYBR® Premix ExTaq™ (Takara Biotech (Dalian), China) on an ABI Applied Biosystems StepOnePlus machine (Life Tech Co., USA). Transcript levels were calculated as relative expression to *SleIF4*α*6*, using the 2^-ΔΔCt^ method, where ΔΔCt = Ct (gene)-Ct (*SleIF4α6*). (Xiao et al. 2008; Xiao et al. 2009).

### Identification of gene co-expressing with *SlPZF1* and gene ontology analysis

Tissue- and cell type- related expression of *SlPZF1* and *PZFI2* was based on in silico analysis of RNA-seq data (http://tea.solgenomics.net/overview), which RNA-seq analysis was coupled with laser capture microdissection (LCM) to produce tissue- and cell type- specific transcriptome data for the cultivated tomato M82 (Shinozaki et al. 2018). Genes co-expressing with *SlPZF2* were selected by default cutoff of correlation coefficient r ≥ 0.7.

Overrepresentation test of GO-slim terms in the list of genes co-expressing with *SlPZF1* was done by PATHER (Protein Analysis Through Evolutionary Relationships, http://pantherdb.org) using the Benjamini-Hochberg False Discovery Rate (FDR) correction (Mi et al. 2018).

### GUS staining

Young seedlings germinated on water-moistened Whatman papers, flower buds, anthesis flowers and fruits at 5, 10, 20 and 30 DPA from *pSlPZF1::GUS* lines were harvested and incubated in 50 mM sodium phosphate buffer (pH 7.0) containing 0.9 mM 5-bromo-4-chloro-3-indolyl-b-glucuronide (X-Gluc), 10 mM EDTA, 0.1% (v/v) Triton X-100 at 37 °C for 2 h. Then, the stained samples were immersed in 70% ethanol at 37 °C for 2 d to remove chlorophyll. Images were captured using a dissection microscope (Leica M125/DFC 420, Germany).

### Flow cytometry

Whole fruits (5 DPA) or pericarp only (10, 20, 30 DPA) collected from 3 to 5 plants for *SlPZF1* OE, RNAi or wild type were chopped gently with a razor blade in Galbraith’s extraction buffer (Galbraith et al. 1983) containing 5 mM sodium metabisulfite in a ratio of 100 mg pericarp per 1 ml extraction buffer. Then, the suspension was filtered twice through 48 μm nylon mesh and stained by DAPI (4′,6-Diamidino-2-phenyindole, Sigma-Aldrich) at a final concentration of 5 μg/ml. The filtrates were analyzed on a flow cytometer (MoFlo™ XDP, Beckman) and data were analyzed with the Beckman Coulter software (Beckman Coulter, USA). Ploidy level of floral sepal cells at anthesis were used as a reference to determine 2C nuclei in the pericarp cells (Cheniclet et al. 2005). Endoreduplication indices (EI) were calculated based on the weighted percentage of nuclei with the DNA content defined: EI = 2C% × 0 + 4C% × 1 + 8C% × 2 + 16C% × 3 + 32C% × 4 + 64C% × 5 (Barow and Meister 2003).

### Yeast two hybrid

A cDNA library for yeast two hybrid screening was prepared from pooled total RNA of Heinz1706 fruits at various development stages (0, 5, 10, 20 and 30 DPA, breaker, red ripe) using Make Your Own “Mate & Plate” Library System (Cat. No. 630490, Takara). A CHROMA SPIN TE-400 Column was used for size-selection of ds cDNA (> 200 bp). For screening proteins interacting with SlPZF1, full-length *SlPZF1* cDNA was amplified from the cDNA library and subcloned into the pGBKT7 DNA-BD cloning vector (pGBKT7-BD-SlPZF1). Yeast two hybrid screening was performed following the procedures described in the Matchmaker Gold Yeast Two-Hybrid System (Cat. No. 630489, Takara). Briefly, after mating between Y2HGold[pGBKT7-BD-SlPZF1] and the library strain Y187 was completed, putative interactions were selected on triple dropout medium SD/−His/−Leu/−Trp for 3–5 days at 30 °C. False positive interactions were discarded if the rescued library plasmids activated one of the three Gal4-responsive reporters *HIS3, ADE2* and *MEL1* in the absence of the bait pGBKT7-BD-SlPZF1.

### Subcellular localization of SlPZF1 and BiFC

For subcellular localization analysis, PCR-amplified full length cDNA of *SlPZF1, PZFI2, 4, 6, 7* and *14* was fused in frame with YFP coding sequence at its C-terminal in the vector pHX64, which the expression of the YFP-SlPZF1 fusion protein was driven by 2x35S promoter. For BiFC assay, full-length SlPZF1 was fused to the N-terminal half of YFP (1-173aa) in the binary vector pHX61, while the five PZFIs (PZFI2, PZFI4, PZFI6, PZFI7 and PZFI14) were respectively fused to the C-terminal half of YFP (174-239aa) in the binary vector pHX62. Both expression cassettes of YFP fusion protein in pHX61 and pHX62 were driven by 2x35S promoter. These plasmids were introduced into *A.tumefaciens* strain GV3101. Subcellular localization and protein interactions between SlPZF1 and PZFIs in the epidermal cells of *Nicotiana benthamiana* leaves were examined after 2 days post infiltration using a confocal scanning microscopy (Zeiss LSM510 Meta, Germany) (Tsai et al. 2005). The excitation and emission for YFP detection were 514 nm and 520-550 nm.

### Supplementary Information

**Additional file 1: Figure S1.** Expression changes of cyclin genes in the whole fruits by altered SlPZF1 expression. **Figure S2.** Co-expression analysis of SlPZF1 during fruit development. **Figure S3.** Merged images showing subcellular localization of SlPZF1 and PZFIs and the interactions between them. **Supplementary Table S1.** Primers used in this study.

## Data Availability

All data generated or analyzed during this study are included in this published article and its supplementary information files. Materials generated in this study are available from corresponding author (H.X.) upon request.

## References

[CR1] Anastasiou E, Kenz S, Gerstung M, MacLean D, Timmer J, Fleck C, Lenhard M (2007). Control of plant organ size by KLUH/CYP78A5-dependent intercellular signaling. Dev Cell.

[CR2] Bailey TL, Boden M, Buske FA, Frith M, Grant CE, Clementi L, Ren J, Li WW, Noble WS (2009). MEME SUITE: tools for motif discovery and searching. Nucleic Acids Res.

[CR3] Baloban M, Vanstraelen M, Tarayre S, Reuzeau C, Cultrone A, Mergaert P, Kondorosi E (2013). Complementary and dose-dependent action of AtCCS52A isoforms in endoreduplication and plant size control. New Phytol.

[CR4] Barow M, Meister A (2003). Endopolyploidy in seed plants is differently correlated to systematics, organ, life strategy and genome size. Plant Cell Environ.

[CR5] Chakrabarti M, Zhang N, Sauvage C, Munos S, Blanca J, Canizares J, Diez MJ, Schneider R, Mazourek M, McClead J, Causse M, van der Knaap E (2013). A cytochrome P450 regulates a domestication trait in cultivated tomato. Proc Natl Acad Sci U S A.

[CR6] Chen L, Cheng JC, Castle L, Sung ZR (1997). EMF genes regulate Arabidopsis inflorescence development. Plant Cell.

[CR7] Cheniclet C, Rong WY, Causse M, Frangne N, Bolling L, Carde JP, Renaudin JP (2005). Cell expansion and endoreduplication show a large genetic variability in pericarp and contribute strongly to tomato fruit growth. Plant Physiol.

[CR8] Chevalier C, Bourdon M, Pirrello J, Cheniclet C, Gevaudant F, Frangne N. Endoreduplication and fruit growth in tomato: evidence in favour of the karyoplasmic ratio theory. J Exp Bot. 2013. 10.1093/jxb/ert1366. 10.1093/jxb/ert366 24187421

[CR9] Chevalier C, Nafati M, Mathieu-Rivet E, Bourdon M, Frangne N, Cheniclet C, Renaudin JP, Gevaudant F, Hernould M (2011). Elucidating the functional role of endoreduplication in tomato fruit development. Ann Bot.

[CR10] Chu YH, Jang JC, Huang Z, van der Knaap E (2019). Tomato locule number and fruit size controlled by natural alleles of lc and fas. Plant Direct.

[CR11] Clay NK, Nelson T (2005). The recessive epigenetic swellmap mutation affects the expression of two step II splicing factors required for the transcription of the cell proliferation gene STRUWWELPETER and for the timing of cell cycle arrest in the Arabidopsis leaf. Plant Cell.

[CR12] Cong B, Tanksley SD (2006). FW2.2 and cell cycle control in developing tomato fruit: a possible example of gene co-option in the evolution of a novel organ. Plant Mol Biol.

[CR13] Czerednik A, Busscher M, Bielen BAM, Wolters-Arts M, de Maagd RA, Angenent GC (2012). Regulation of tomato fruit pericarp development by an interplay between CDKB and CDKA1 cell cycle genes. J Exp Bot.

[CR14] De Veylder L, Beeckman T, Beemster GT, Krols L, Terras F, Landrieu I, van der Schueren E, Maes S, Naudts M, Inze D (2001). Functional analysis of cyclin-dependent kinase inhibitors of Arabidopsis. Plant Cell.

[CR15] De Veylder L, Larkin JC, Schnittger A (2011). Molecular control and function of endoreplication in development and physiology. Trends Plant Sci.

[CR16] Dewitte W, Riou-Khamlichi C, Scofield S, Healy JM, Jacqmard A, Kilby NJ, Murray JA (2003). Altered cell cycle distribution, hyperplasia, and inhibited differentiation in Arabidopsis caused by the D-type cyclin CYCD3. Plant Cell.

[CR17] Dewitte W, Scofield S, Alcasabas AA, Maughan SC, Menges M, Braun N, Collins C, Nieuwland J, Prinsen E, Sundaresan V, Murray JA (2007). Arabidopsis CYCD3 D-type cyclins link cell proliferation and endocycles and are rate-limiting for cytokinin responses. Proc Natl Acad Sci U S A.

[CR18] Englbrecht CC, Schoof H, Bohm S (2004). Conservation, diversification and expansion of C2H2 zinc finger proteins in the Arabidopsis thaliana genome. BMC Genomics.

[CR19] Fox DT, Duronio RJ (2012). Endoreplication and polyploidy: insights into development and disease. Development.

[CR20] Frary A, Nesbitt TC, Grandillo S, Knaap E, Cong B, Liu J, Meller J, Elber R, Alpert KB, Tanksley SD (2000). fw2.2: a quantitative trait locus key to the evolution of tomato fruit size. Science.

[CR21] Galbraith DW, Harkins KR, Maddox JM, Ayres NM, Sharma DP, Firoozabady E (1983). Rapid flow cytometric analysis of the cell cycle in intact plant tissues. Science.

[CR22] Gendall AR, Levy YY, Wilson A, Dean C (2001). The VERNALIZATION 2 gene mediates the epigenetic regulation of vernalization in Arabidopsis. Cell.

[CR23] Gillaspy G, Ben-David H, Gruissem W (1993). Fruits: a developmental perspective. Plant Cell.

[CR24] Gomez E, Royo J, Guo Y, Thompson R, Hueros G (2002). Establishment of cereal endosperm expression domains: identification and properties of a maize transfer cell-specific transcription factor, ZmMRP-1. Plant Cell.

[CR25] Gonzalez N, Gevaudant F, Hernould M, Chevalier C, Mouras A (2007). The cell cycle-associated protein kinase WEE1 regulates cell size in relation to endoreduplication in developing tomato fruit. Plant J.

[CR26] Grigg SP, Canales C, Hay A, Tsiantis M (2005). SERRATE coordinates shoot meristem function and leaf axial patterning in Arabidopsis. Nature.

[CR27] Hayashi K, Hasegawa J, Matsunaga S (2013). The boundary of the meristematic and elongation zones in roots: endoreduplication precedes rapid cell expansion. Sci Rep.

[CR28] Heyman J, De Veylder L (2012). The anaphase-promoting complex/cyclosome in control of plant development. Mol Plant.

[CR29] Hirano T, Matsuzawa T, Takegawa K, Sato MH (2011). Loss-of-function and gain-of-function mutations in FAB1A/B impair endomembrane homeostasis, conferring pleiotropic developmental abnormalities in Arabidopsis. Plant Physiol.

[CR30] Hisanaga T, Kawade K, Tsukaya H (2015). Compensation: a key to clarifying the organ-level regulation of lateral organ size in plants. J Exp Biol.

[CR31] Imai KK, Ohashi Y, Tsuge T, Yoshizumi T, Matsui M, Oka A, Aoyama T (2006). The A-type cyclin CYCA2;3 is a key regulator of ploidy levels in Arabidopsis endoreduplication. Plant Cell.

[CR32] Inagaki S, Umeda M (2011). Cell-cycle control and plant development. Int Rev Cell Mol Biol.

[CR33] Inze D, De Veylder L (2006). Cell cycle regulation in plant development. Annu Rev Genet.

[CR34] Jegu T, Latrasse D, Delarue M, Mazubert C, Bourge M, Hudik E, Blanchet S, Soler MN, Charon C, De Veylder L, Raynaud C, Bergounioux C, Benhamed M (2013). Multiple functions of kip-related protein5 connect endoreduplication and cell elongation. Plant Physiol.

[CR35] Joubes J, Walsh D, Raymond P, Chevalier C (2000). Molecular characterization of the expression of distinct classes of cyclins during the early development of tomato fruit. Planta.

[CR36] Kerschen A, Napoli CA, Jorgensen RA, Muller AE (2004). Effectiveness of RNA interference in transgenic plants. FEBS Lett.

[CR37] Kumar N, Harashima H, Kalve S, Bramsiepe J, Wang K, Sizani BL, Bertrand LL, Johnson MC, Faulk C, Dale R, Simmons LA, Churchman ML, Sugimoto K, Kato N, Dassanayake M, Beemster G, Schnittger A, Larkin JC (2015). Functional conservation in the SIAMESE-RELATED family of cyclin-dependent kinase inhibitors in land plants. Plant Cell.

[CR38] Kuroda TS, Maita H, Tabata T, Taira T, Kitaura H, Ariga H, Iguchi-Ariga SM (2004). A novel nucleolar protein, PAPA-1, induces growth arrest as a result of cell cycle arrest at the G1 phase. Gene.

[CR39] Leiva-Neto JT, Grafi G, Sabelli PA, Dante RA, Woo YM, Maddock S, Gordon-Kamm WJ, Larkins BA (2004). A dominant negative mutant of cyclin-dependent kinase a reduces endoreduplication but not cell size or gene expression in maize endosperm. Plant Cell.

[CR40] Liu Y, Ye W, Li B, Zhou X, Cui Y, Running MP, Liu K (2012). CCS52A2/FZR1, a cell cycle regulator, is an essential factor for shoot apical meristem maintenance in Arabidopsis thaliana. BMC Plant Biol.

[CR41] Luo M, Bilodeau P, Koltunow A, Dennis ES, Peacock WJ, Chaudhury AM (1999). Genes controlling fertilization-independent seed development in Arabidopsis thaliana. Proc Natl Acad Sci U S A.

[CR42] Mathieu-Rivet E, Gevaudant F, Sicard A, Salar S, Do PT, Mouras A, Fernie AR, Gibon Y, Rothan C, Chevalier C, Hernould M (2010). Functional analysis of the anaphase promoting complex activator CCS52A highlights the crucial role of endo-reduplication for fruit growth in tomato. Plant J.

[CR43] McCormick S, Lindsey K (1991). Transformation of tomato with agrobacterium tumefaciens. Plant tissue culture manual: fundamentals and applications.

[CR44] Mi H, Muruganujan A, Ebert D, Huang X, Thomas PD (2018). PANTHER version 14: more genomes, a new PANTHER GO-slim and improvements in enrichment analysis tools. Nucleic Acids Res.

[CR45] Mizukami Y, Fischer RL (2000). Plant organ size control: <em>AINTEGUMENTA</em> regulates growth and cell numbers during organogenesis. Proc Natl Acad Sci U S A.

[CR46] Mu Q, Huang Z, Chakrabarti M, Illa-Berenguer E, Liu X, Wang Y, Ramos A, van der Knaap E (2017). Fruit weight is controlled by cell size regulator encoding a novel protein that is expressed in maturing tomato fruits. PLoS Genet.

[CR47] Munos S, Ranc N, Botton E, Berard A, Rolland S, Duffe P, Carretero Y, Le Paslier MC, Delalande C, Bouzayen M, Brunel D, Causse M (2011). Increase in tomato locule number is controlled by two single-nucleotide polymorphisms located near WUSCHEL. Plant Physiol.

[CR48] Ohashi-Ito K, Bergmann DC (2007). Regulation of the Arabidopsis root vascular initial population by LONESOME HIGHWAY. Development.

[CR49] Ohashi-Ito K, Oguchi M, Kojima M, Sakakibara H, Fukuda H (2013). Auxin-associated initiation of vascular cell differentiation by LONESOME HIGHWAY. Development.

[CR50] Royo J, Gomez E, Barrero C, Muniz LM, Sanz Y, Hueros G (2009). Transcriptional activation of the maize endosperm transfer cell-specific gene BETL1 by ZmMRP-1 is enhanced by two C2H2 zinc finger-containing proteins. Planta.

[CR51] Sablowski R (2016). Coordination of plant cell growth and division: collective control or mutual agreement?. Curr Opin Plant Biol.

[CR52] Sablowski R, Carnier Dornelas M (2014). Interplay between cell growth and cell cycle in plants. J Exp Bot.

[CR53] Shinozaki Y, Nicolas P, Fernandez-Pozo N, Ma Q, Evanich DJ, Shi Y, Xu Y, Zheng Y, Snyder SI, Martin LBB, Ruiz-May E, Thannhauser TW, Chen K, Domozych DS, Catalá C, Fei Z, Mueller LA, Giovannoni JJ, Rose JKC (2018). High-resolution spatiotemporal transcriptome mapping of tomato fruit development and ripening. Nat Commun.

[CR54] Smet W, Sevilem I, de Luis Balaguer MA, Wybouw B, Mor E, Miyashima S, Blob B, Roszak P, Jacobs TB, Boekschoten M, Hooiveld G, Sozzani R, Helariutta Y, De Rybel B (2019). DOF2.1 controls Cytokinin-dependent vascular cell proliferation downstream of TMO5/LHW. Curr Biol.

[CR55] Sterken R, Kiekens R, Boruc J, Zhang F, Vercauteren A, Vercauteren I, De Smet L, Dhondt S, Inze D, De Veylder L, Russinova E, Vuylsteke M (2012). Combined linkage and association mapping reveals CYCD5;1 as a quantitative trait gene for endoreduplication in Arabidopsis. Proc Natl Acad Sci U S A.

[CR56] Sun H, Qian Q, Wu K, Luo J, Wang S, Zhang C, Ma Y, Liu Q, Huang X, Yuan Q, Han R, Zhao M, Dong G, Guo L, Zhu X, Gou Z, Wang W, Wu Y, Lin H, Fu X (2014). Heterotrimeric G proteins regulate nitrogen-use efficiency in rice. Nat Genet.

[CR57] Tamura K, Peterson D, Peterson N, Stecher G, Nei M, Kumar S (2011). MEGA5: molecular evolutionary genetics analysis using maximum likelihood, evolutionary distance, and maximum parsimony methods. Mol Biol Evol.

[CR58] Tanksley SD (2004). The genetic, developmental, and molecular bases of fruit size and shape variation in tomato. Plant Cell.

[CR59] Thompson DS (2005). How do cell walls regulate plant growth?. J Exp Bot.

[CR60] Tsai CW, Redinbaugh MG, Willie KJ, Reed S, Goodin M, Hogenhout SA (2005). Complete genome sequence and in planta subcellular localization of maize fine streak virus proteins. J Virol.

[CR61] Van den Heuvel KJ, Van Lipzig RH, Barendse GW, Wullems GJ (2002). Regulation of expression of two novel flower-specific genes from tomato (Solanum lycopersicum) by gibberellin. J Exp Bot.

[CR62] Vanneste S, Coppens F, Lee E, Donner TJ, Xie Z, Van Isterdael G, Dhondt S, De Winter F, De Rybel B, Vuylsteke M, De Veylder L, Friml J, Inze D, Grotewold E, Scarpella E, Sack F, Beemster GT, Beeckman T (2011). Developmental regulation of CYCA2s contributes to tissue-specific proliferation in Arabidopsis. EMBO J.

[CR63] Vera-Sirera F, De Rybel B, Úrbez C, Kouklas E, Pesquera M, Álvarez-Mahecha JC, Minguet EG, Tuominen H, Carbonell J, Borst JW, Weijers D, Blázquez MA (2015). A bHLH-based feedback loop restricts vascular cell proliferation in plants. Dev Cell.

[CR64] Weingartner M, Criqui MC, Meszaros T, Binarova P, Schmit AC, Helfer A, Derevier A, Erhardt M, Bogre L, Genschik P (2004). Expression of a nondegradable cyclin B1 affects plant development and leads to endomitosis by inhibiting the formation of a phragmoplast. Plant Cell.

[CR65] Xiao H, Jiang N, Schaffner E, Stockinger EJ, van der Knaap E (2008). A retrotransposon-mediated gene duplication underlies morphological variation of tomato fruit. Science.

[CR66] Xiao H, Radovich C, Welty N, Hsu J, Li D, Meulia T, van der Knaap E (2009). Integration of tomato reproductive developmental landmarks and expression profiles, and the effect of SUN on fruit shape. BMC Plant Biol.

[CR67] Xiao H, Wang Y, Liu D, Wang W, Li X, Zhao X, Xu J, Zhai W, Zhu L (2003). Functional analysis of the rice AP3 homologue OsMADS16 by RNA interference. Plant Mol Biol.

[CR68] Xu C, Liberatore KL, MacAlister CA, Huang Z, Chu YH, Jiang K, Brooks C, Ogawa-Ohnishi M, Xiong G, Pauly M, Van Eck J, Matsubayashi Y, van der Knaap E, Lippman ZB (2015). A cascade of arabinosyltransferases controls shoot meristem size in tomato. Nat Genet.

[CR69] Yoshizumi T, Tsumoto Y, Takiguchi T, Nagata N, Yamamoto YY, Kawashima M, Ichikawa T, Nakazawa M, Yamamoto N, Matsui M (2006). Increased level of polyploidy1, a conserved repressor of CYCLINA2 transcription, controls endoreduplication in Arabidopsis. Plant Cell.

[CR70] Zielke N, Edgar BA, DePamphilis ML (2013). Endoreplication. Cold Spring Harb Perspect Biol.

