# Supplementary material for: Fruit size control by a zinc finger protein regulating pericarp cell size in tomato
Source: Mol Hortic. 2021 Aug 14;1:6. doi: 10.1186/s43897-021-00009-6 (PMC10515234; doi:10.1186/s43897-021-00009-6)
Supplement: Supplementary file 1 — Additional file 1: Figure S1. Expression changes of cyclin genes in the whole fruits by altered SlPZF1 expression. Figure S2. Co-expression analysis of SlPZF1 during fruit development. Figure S3. Merged images showing subcellular localization of SlPZF1 and PZFIs and the interactions between them. Supplementary Table S1. Primers used in this study. [file 43897_2021_9_MOESM1_ESM.pdf]

Supplementary materials

Fruit size control by a zinc finger protein regulating pericarp cell size in tomato

Fangfang Zhao[1,2,#], Jiajing Zhang[1,3,#], Lin Weng[1], Meng Li[1] , Quanhua Wang[3], and Han Xiao[1,*]

Supplemntary materials include:

Supplementary Figure S1-3

Supplementary Table S1

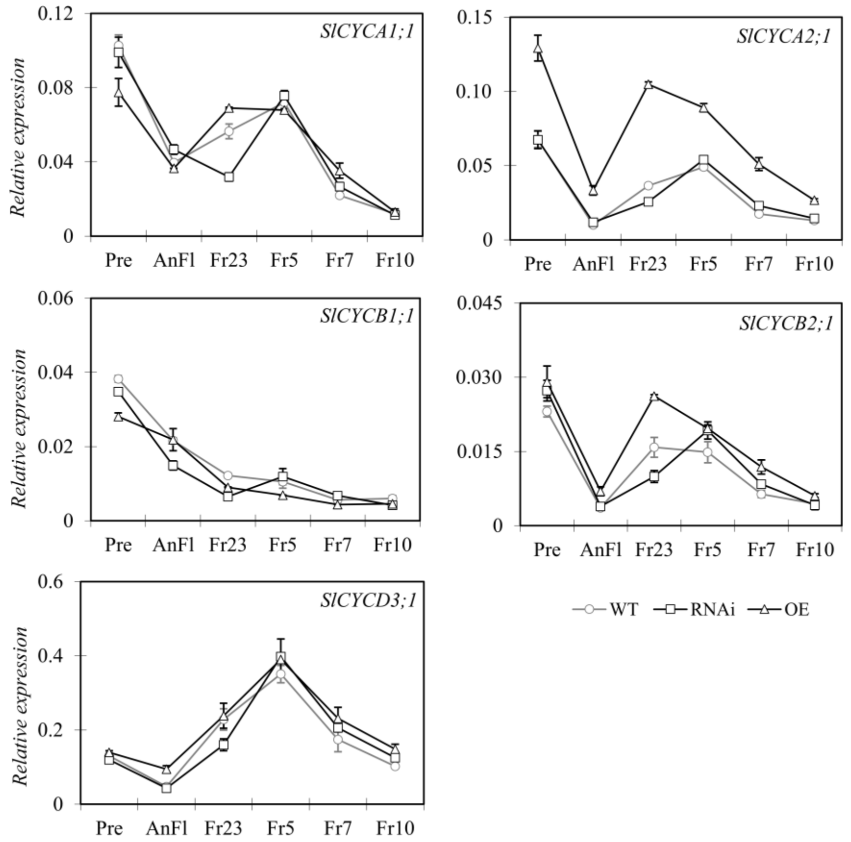

Figure S1. expression changes of cyclin genes in the whole fruits by altered *SlPZF1* expression

Total RNA was extracted from pooled samples of three plants of the RNAi, OE and wild type at the same developmental stages. Pre, young flower buds preanthesis; AnFl, anthesis flowers; Fr2/3, fruits at 2 and 3 DPA; Fr5, Fr7 and Fr10 are fruits at 5, 7 and 10 DPA, respectively. Relative expression level was normalized to *SleIF4a6* and data are means ± sd. n=3.

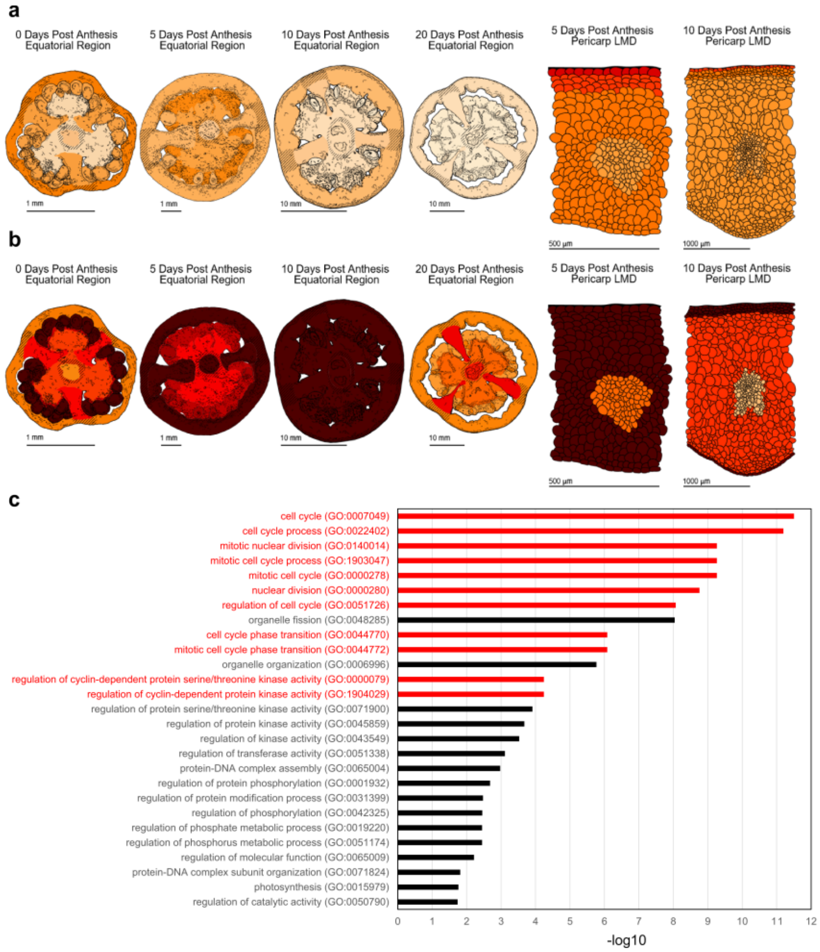

Figure S2. Co-expression analysis of SlPZF1 during fruit development

a, expression pattern of *SlPZF1* in M82 fruits. **b**, expression pattern of *PZFI2* in M82 fruits. **c**, gene ontology analysis of genes co-expressing with *SlPZF1* during fruit development. Enriched GO-slim terms were identified from the 1137 genes co-expressing with SlPZF1 in M82 fruits (correlation coefficient ≥0.7) by PATHER with a cutoff of fold enrichment >2 and FDR <0.05 (http://go.pantherdb.org/). The images in (**a, b**) were obtained from TEA (http://tea.solgenomics.net/expression_viewer/).

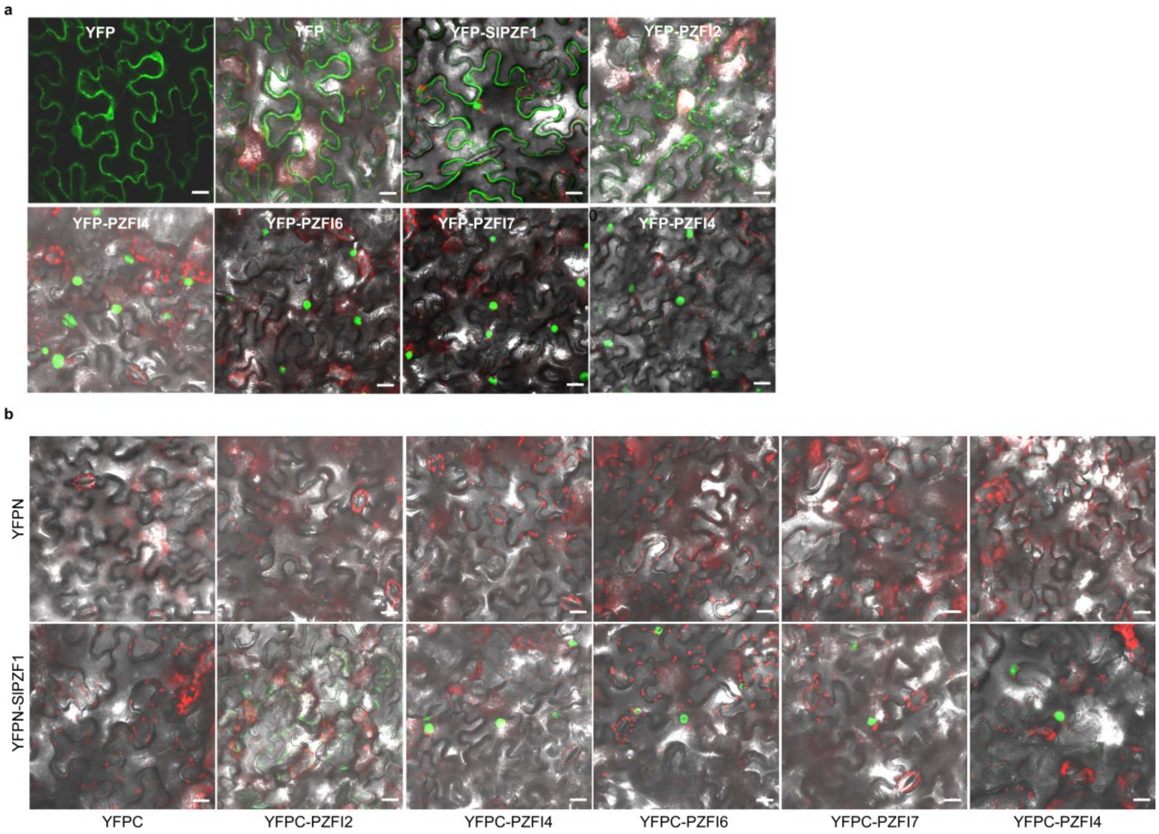

Figure S3. SlPZF1 interacted with PZFIs in *N. benthamiana* leaves.

a, subcellular localization of YFP alone and YFP fused with SlPZF1 and five PZFIs. **b**, BiFC verification of the interactions between SlPZF1 and five PZFIs in *N.benthamiana* leaves. Except YFP alone (**a**), merged images of YFP, autofluorescence and bright field were shown here, the single channel images of YFP signals were presented in Figure 8. YFPN, N-terminal part of YFP; YFPC, C-terminal part of YFP. Scale bar = 20 μm.

Supplementary table 1. Information of primers used in this study

Primer names	Sequence (5'-3')	Genes	Product sizes	Notes
**Primers used for constructing vectors of plant transformation**				
xp0687	GCTCTAGAGCCATGGCAATGGGGGAG AAGA	*SlPZF1*	2754bp	construction of overexpression vector
xp0688	CGAGCTCTTAGGCTTTGCAGATTACCA CAAAACAA			
xp0791	TGCTCTAGAGGCGCGCCGTTATCTGAG TATAAGTAGAGGAAAAG	*SlPZF1*	395bp (-78-317 bp)	RNAi construct
xp0792	CGGGATCCATTTAAATAAACTAGTGAT TTTCAGCTCACATC			
xp1027	CGCGGATCCTTTCAAGATTCTATCTTTT CAACAAC	*SlPZF1*	2300bp	pSlPZF1::GUS
xp1073	CCGGAATTCATCATACACAAGTTGAGT AGCAGATC			
**Primers used for genotyping**				
xp0515	CTACACAGCCATCGGTCCAG	*HygR*	787bp	
xp0516	CGTTATGTTTATCGGCACTTTG			
xp0517	AAAGCCACGCACATTTAGGA	*BastaR*	957bp	nt 181-1137 on pFGC5941
xp0518	AGATACGCTGACACGCCAAG			
**qRT-PCR primers**				
xp0560	TGGAACTTCTTCTGGGGTCAC	*SlPZF1*	400bp	Solyc07g063970
xp0561	TTCGACAGACGCAGCATTTAT			
xp1163	CTCTGAAGCACCACACTGGA	*SlCYCB1;1*	148bp	Solyc06g073610
xp1164	AAGAGGGCAACAGCACATCT			
xp2491	GGGATGTATTTTTGCCGAGA	*SlCDKB1*	187bp	Solyc10g074720

xp2492	GAACAGCAGAGGCCAAGTTC			
xp2493	GGGAGGGTACCTATGGAAA	*SlCDKB2*	150bp	Solyc04g082840
xp2494	CCCTTGAGAGCATTCTGAGG			
xp2495	CAAGCACACTTCAAGGACCA	*SlCYCA2;1*	168bp	Solyc06g065680
xp2496	AGCCTTCCTGTTTCAAGCAA			
xp2499	CGGTTTCTCATTCCTCCAAA	*SlCYCA1;1*	209bp	Solyc11g005090
xp2500	AAGTGGCAGGAACAGGAATG			
xp2509	CTCCAGCTTGTTGGATTGGT	*SlCYCB2;1)*	218bp	Solyc02g082820
xp2510	GTCACATTGAGCAGCCTTGA			
xp2519	GAGCACACCTTGCAGTTTGA	*SlKRP1*	168bp	Solyc09g091780
xp2520	CTCCTTTTCTGCACGGGTAA			
xp2521	CATGATGAGCTTGCCACACT	*SlCYCD3;1*	206bp	Solyc02g092980
xp2522	CTGAAAGCACCATCCAGACA			
xp2523	ATTTTGGAAGCGCAAAAGTG	*SlCDKA1*	205bp	Solyc10g037910
xp2524	CTCAACAAGCTGGTCCACAA			
xp2533	TCCTCCGTGCATTAGAAACC	*SlWEE1*	170bp	Solyc09g074830
xp2534	TTCCCACTACCGATTTGCTC			
xp2535	GCTCATCCATGCTGTCTTCA	*SlCCS52A*	203bp	Solyc08g080080
xp2536	GCACAGGTTGTGTTGAATGG			
**Primers used for subcellular localization and BiFC**				
XP7094	ggg GGTACC ATGACACAAATCATTCAAAATATGG	PZFI2/Solyc 01g006400	779bp	BiFC
XP7095	aaa GTCGAC CTTCATTCATCATCCTTGGATAGCT			
XP7707	aaaaa GGTACC CTTAATGCAGAAAGACCAAGTTTGA	PZFI4/Solyc 09g072570	1449bp	BiFC
XP7603	aaaaaa GTCGAC			

	TCAGTGAAGGAAGTCCTTCATTGGA			
XP6989	aaaaaa GGTACC ATGTCTAGAGGCTCTGTTTCCAGAG	PZFI6/Solyc 01g079350	1398bp	BiFC
XP6990	aaaaaa GTCGAC TCAAGCCTGCTCCTTCTCACGAATT			
XP6987	aaaaaa GGTACC ATGTACAAATATAAAATGGACCCTT	PZFI7/Solyc 03g116830	1342bp	BiFC
XP7220	aaaaaa GTCGAC CTAGGATCCATACCTTTGTGGCAAG			
XP6985	aaaaaa GGTACC ATGGGGTATTTGCTGAAAGAGGTTT	PZFI14/Soly c11g068960	2793bp	BiFC
XP6986	aaaaaa GGTACC TTAATTACAGGGTCTACTACCTGTT			
XP7094	ggg GGTACC ATGACACAAATCATTCAAAATATGG	PZFI2/Solyc 01g006400	779bp	Subcellular localization
XP7095	aaa GTCGAC CTTCATTCATCATCCTTGGATAGCT			
XP7707	aaaaa GGTACC CTTAATGCAGAAAGACCAAGTTTGA	PZFI4/Solyc 09g072570	1449bp	Subcellular localization
XP7603	aaaaaa GTCGAC TCAGTGAAGGAAGTCCTTCATTGGA			
XP6989	aaaaaa GGTACC ATGTCTAGAGGCTCTGTTTCCAGAG	PZFI6/Solyc 01g079350	1398bp	Subcellular localization
XP6990	aaaaaa GTCGAC TCAAGCCTGCTCCTTCTCACGAATT			
XP6987	aaaaaa GGTACC ATGTACAAATATAAAATGGACCCTT	PZFI7/Solyc 03g116830	1342bp	Subcellular localization
XP7220	aaaaaa GTCGAC CTAGGATCCATACCTTTGTGGCAAG			
XP6985	aaaaaa GGTACC ATGGGGTATTTGCTGAAAGAGGTTT	PZFI14/Soly c11g068960	2793bp	Subcellular localization

XP6986	aaaaaa GGTACC TTAATTACAGGGTCTACTACCTGTT			
XP6991	aaaaaa GGTACC ATGGGGGAGAAGAAGAATAT	PZF1/Solyc07g063970	1284bp	Subcellular localization and BiFC
XP6992	aaaaaa GTCGAC TTAGGCTTTGCAGATTACCACAAAA			
